# Inhibition of Striatal miR-141-3p Ameliorates Cognitive Deficits and Neuroinflammation in an ADHD Animal Model

**DOI:** 10.3390/ph19091461

**Published:** 2026-09-15

**Authors:** Tung-Ming Chang, Chun-Ching Chiu, Yen-Chen Chen, Zhi-Han Wen, Tsai-Ching Hsu, Bor-Show Tzang

**Affiliations:** 1 Pediatric Neurology, Changhua Christian Children’s Hospital, Changhua 500, Taiwan; 128658@cch.org.tw; 2 Department of Post-Baccalaureate Medicine, College of Medicine, National Chung Hsing University, Taichung 402, Taiwan; 3 Institute of Medicine, Chung Shan Medical University, Taichung 402, Taiwan; s31065@gmail.com (C.-C.C.);; 4 Department of Biochemistry, School of Medicine, Chung Shan Medical University, Taichung 402, Taiwan; 5 Department of Biomedical Sciences, College of Medical Science and Technology, Chung Shan Medical University, Taichung 402, Taiwan; 6 Immunology Research Center, Chung Shan Medical University, Taichung 402, Taiwan; 7 Department of Clinical Laboratory, Chung Shan Medical University Hospital, Taichung 402, Taiwan

**Keywords:** attention-deficit/hyperactivity disorder (ADHD), microRNAs (miRNAs), spontaneous hypertensive rats (SHRs), spontaneous alternation

## Abstract

**Background/Objectives**: Attention-deficit/hyperactivity disorder (ADHD) is a common neurodevelopmental disorder characterized by inattention, hyperactivity, and impulsivity that may persist into adulthood. Emerging evidence suggests that microRNAs (miRNAs) contribute to ADHD pathogenesis, but the role of miR-141-3p remains unclear. This study investigated the effects of miR-141-3p inhibition on ADHD-like behaviors in spontaneous hypertensive rats (SHRs). **Methods**: We used RT-qPCR, immunoblotting, immunohistochemistry, ELISA, and a Y-maze test to assess molecular and behavioral changes after striatal stereotaxic injection of an miR-141-3p antagomir (AT). **Results**: Striatal miR-141-3p expression was significantly higher in SHRs than in WKY rats, while taurine treatment reduced its expression. Inhibition of miR-141-3p suppressed inflammasome-related signaling pathways, including the TLR4/NF-κB/NLRP3 and TNF-α/NF-κB/NLRP3 pathways. It also reduced the number of NLRP3-positive cells and the levels of IL-1β, IL-18, and IL-17A in the striatum and improved spontaneous alternation performance in the Y-maze. **Conclusions**: These findings suggest that miR-141-3p may contribute to ADHD-like pathology through neuroinflammatory mechanisms and warrants further investigation as a potential therapeutic target.

## 1. Introduction

Attention-deficit/hyperactivity disorder (ADHD) is known as a prevalent neurodevelopmental disorder in children that can continue into adulthood [1]. The diagnosis of ADHD is primarily based on the criteria outlined in the Diagnostic and Statistical Manual of Mental Disorders, Fifth Edition (DSM-5), published by the American Psychiatric Association [2]. Approximately 3–7% of children worldwide suffer from ADHD, with key symptoms like inattention, hyperactivity, and impulsivity [3]. Current treatment for ADHD primarily involves medications, including central nervous system stimulants like methylphenidate and non-stimulant drugs like atomoxetine [4]. Alongside medication, behavioral therapies such as cognitive-behavioral therapy, reward-based systems to encourage positive behaviors, education, support, and structured daily routines also play a critical role in managing ADHD [5,6,7]. Although the etiology of ADHD remains incompletely understood, researchers have identified several contributing factors, including genetic, neurophysiological, and environmental influences [8,9].

MicroRNAs (miRNAs) are small RNA molecules about 22 nucleotides long. They can act by binding to the 3′ untranslated region (3′ UTR) of target mRNAs, thereby inhibiting mRNA translation [10,11]. Evidence indicates that miRNAs play pivotal roles in various biological processes, including cell proliferation, differentiation, apoptosis, neural development, and synaptic plasticity [12,13]. Given that genetic abnormalities are a major contributor to ADHD, it is likely that certain miRNAs, such as miR-34a and miR-132, are expressed differently in ADHD patients compared to non-ADHD individuals [14,15,16]. In addition, a comparative study of 52 ADHD patients and 52 healthy volunteers reported that the levels of miRNAs 18a-5p, 22-3p, 24-3p, 106b-5p, and 107 were significantly decreased in ADHD patients, whereas miRNA 155a-5p was considerably elevated [17]. Another larger study recruiting 754 ADHD patients and 766 controls also reported that miR-34c-3p was overexpressed in peripheral blood monocytes in ADHD patients [18]. Follow-up studies have also confirmed that many of these miRNAs are associated with genes that regulate neurotransmission, synaptic function, and neural development, thereby affecting synaptic plasticity and neurotransmitter activity, which in turn influence attention and behavioral control [12,13,19].

The spontaneously hypertensive rat (SHR) has been widely regarded as a valid animal model of attention-deficit/hyperactivity disorder (ADHD) because it displays core behavioral features of ADHD, including hyperactivity, inattention, and impulsivity, which emerge early in development and persist into adulthood. Neurobiologically, SHRs show dysfunctions in dopaminergic and noradrenergic signaling, particularly within frontostriatal circuits that are critical for attention and executive control, paralleling abnormalities observed in individuals with ADHD. Additionally, ADHD medications such as methylphenidate and amphetamine reduce hyperactivity and improve attentional performance in SHRs, demonstrating pharmacological relevance and predictive validity. Together, these behavioral, neurochemical, and treatment-response characteristics support the SHR as a robust model for studying ADHD development [20,21].

Taurine plays critical roles in regulating neurodevelopment and neurotransmission in the brain and exerts protective effects in treating various conditions, including skeletal myopathies, metabolic syndromes, cardiovascular diseases, and central nervous system disorders [22,23,24,25]. Previous evidence has indicated that taurine has beneficial effects on ADHD-like symptoms in SHRs by decreasing locomotor activity [26], mALFF signals in the bilateral hippocampus [27], dopamine uptake in striatal synaptosomes [28], and inflammatory factors [27,29]. Indeed, the cause of inflammation is complex and has been strongly linked to the etiology of ADHD [30,31]. Certain inflammatory cytokines, specifically interleukin-1 (IL-1), IL-17, IL-18, and tumor necrosis factor (TNF), play crucial roles in the pathological development of ADHD [32,33]. Interestingly, miR-141-3p, a key regulator in brain inflammation and memory deficit [34], was found to be downregulated in the striatum of SHRs with taurine supplementation in our recent test. Therefore, we investigated the role of miR-141-3p in ADHD-like symptoms in SHRs and the possible underlying mechanisms.

## 2. Results

### 2.1. The Effects of Taurine and miR-141-3p Antagomir on miR-141-3p Expression in the Striatum of SHRs

**Figure 1:**
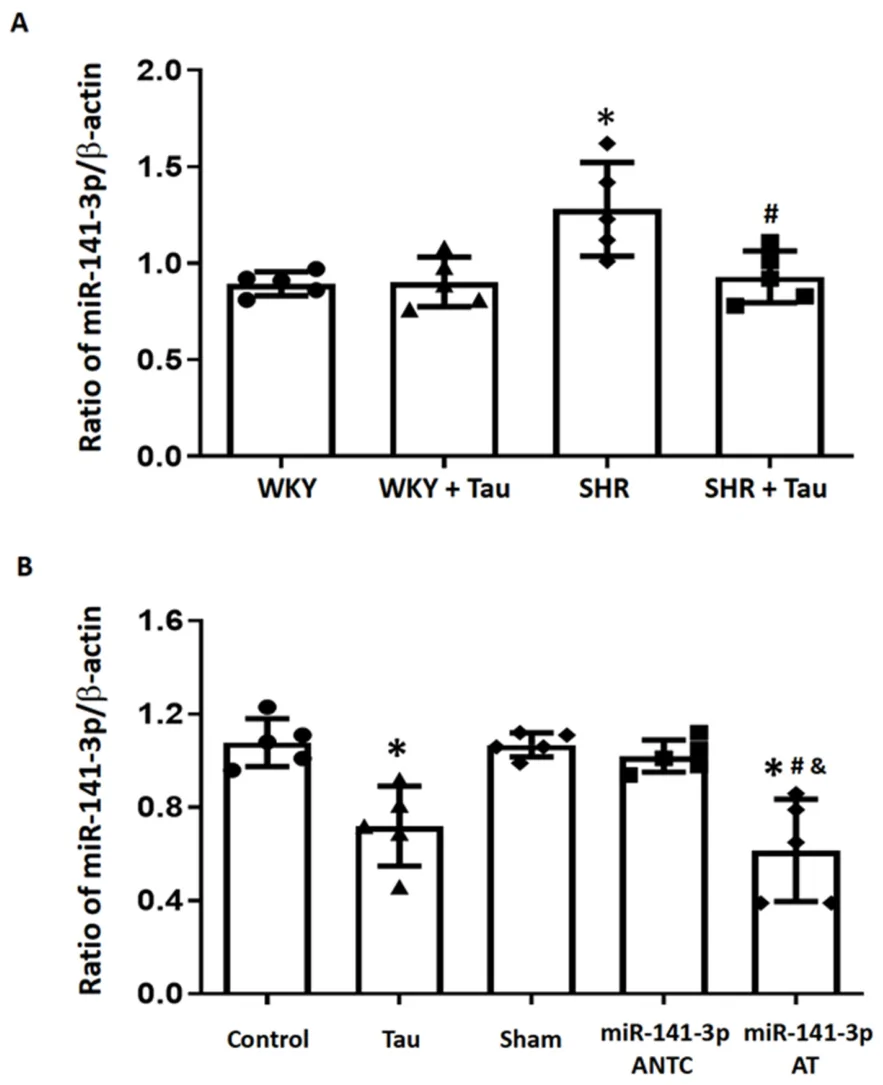
The expression of miR-141-3p in the striatum of WKY rats and SHRs. (**A**) The ratio of miR-141-3p level relative to β-actin in the striatum of WKY rats and SHRs fed with a control diet or a taurine (Tau) diet. Data are represented as mean ± S.D. The marks * and # indicate significant differences compared to the WKY and SHR groups. (**B**) The ratio of miR-141-3p level relative to β-actin in the striatum of SHRs with different treatments. The symbols *, #, and & indicate significant differences compared with the Control, Sham, and miR-141-3p ATNC groups, respectively.

We evaluated and compared miR-141-3p expression in the striatum of WKY rats and SHRs using RT-qPCR. As shown in Figure 1A, no significant difference in the striatal miR-141-3p level was detected between the WKY rats fed a taurine diet and those fed a control diet. Compared with WKY rats fed a control diet, miR-141-3p levels in the striatum of SHRs fed a control diet were significantly elevated. Notably, a significantly decreased miR-141-3p level was observed in the striatum of SHRs fed a taurine diet compared to those fed a control diet (Figure 1A). Consistently, a significantly lower miR-141-3p level was detected in the striatum of SHRs fed a taurine diet and those with striatal stereotaxic injection of miR-141-3p antagomir (AT) compared to those from the Control, Sham, and miR-141-3p antagomir negative control (ATNC) groups, respectively (Figure 1B).

### 2.2. Administration of miR-141-3p Antagomir Reduces Inflammasome-Related Signaling

**Figure 2:**
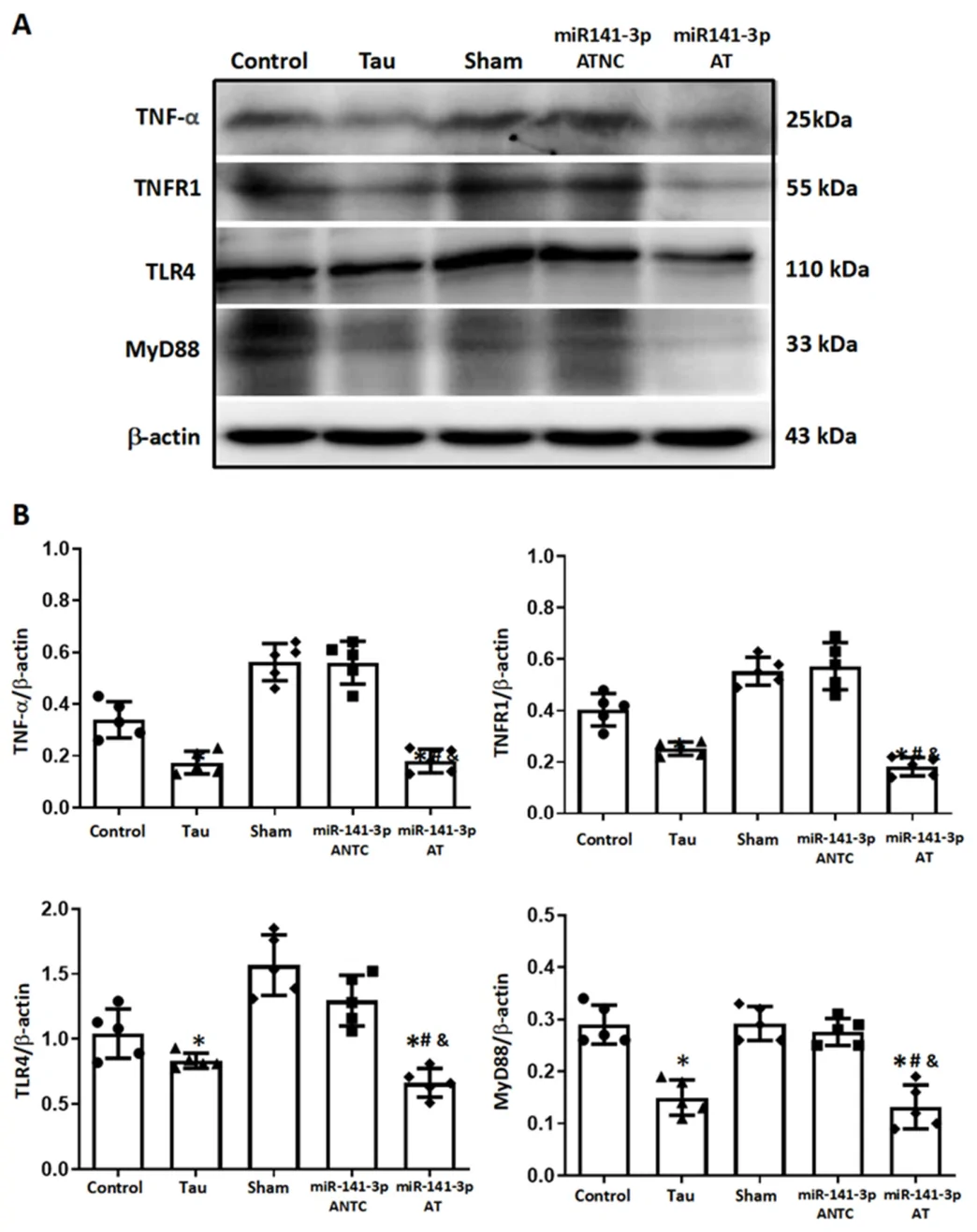
The levels of inflammatory proteins in the striatum of SHRs. (**A**) Expression of TNF-α, TNFR1, TLR4, and MyD88 proteins in the striatum of SHRs subjected to different treatments. (**B**) Relative protein levels of TNF-α, TNFR1, TLR4, and MyD88 normalized to β-actin. Data are presented as the mean ± S.D. The symbols *, #, and & indicate significant differences compared with the Control, Sham, and miR-141-3p ATNC groups, respectively. Control: fed the Cho diet; Tau: fed 45 mM taurine; Sham: fed the Cho diet; miR ATNC: injected with the miR-141-3p antagomir negative control; miR AT: injected with the miR-141-3p antagomir.

**Figure 3:**
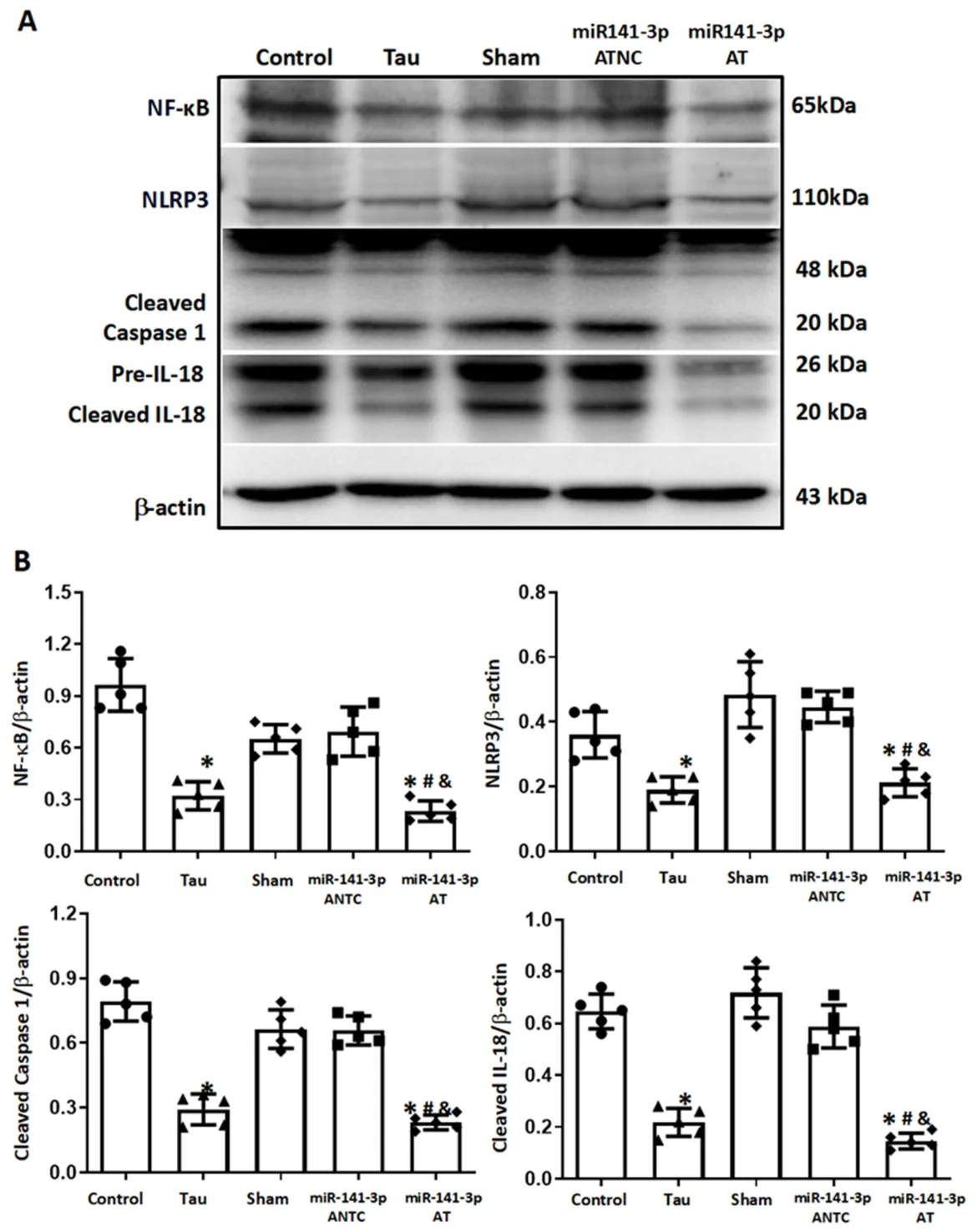
The levels of inflammasome-related proteins in the striatum of SHRs. (**A**) Expression of NF-κB, NLRP3, caspase-1, and IL-18 proteins in the striatum of SHRs subjected to different treatments. (**B**) Relative protein levels of NF-κB, NLRP3, caspase-1, and IL-18 normalized to β-actin. Data are presented as the mean ± S.D. The symbols *, #, and & indicate significant differences compared with the Control, Sham, and miR-141-3p ATNC groups, respectively. Control: fed the Cho diet; Tau: fed 45 mM taurine; Sham: fed the Cho diet; miR ATNC: injected with the miR-141-3p antagomir negative control; miR AT: injected with the miR-141-3p antagomir.

**Figure 4:**
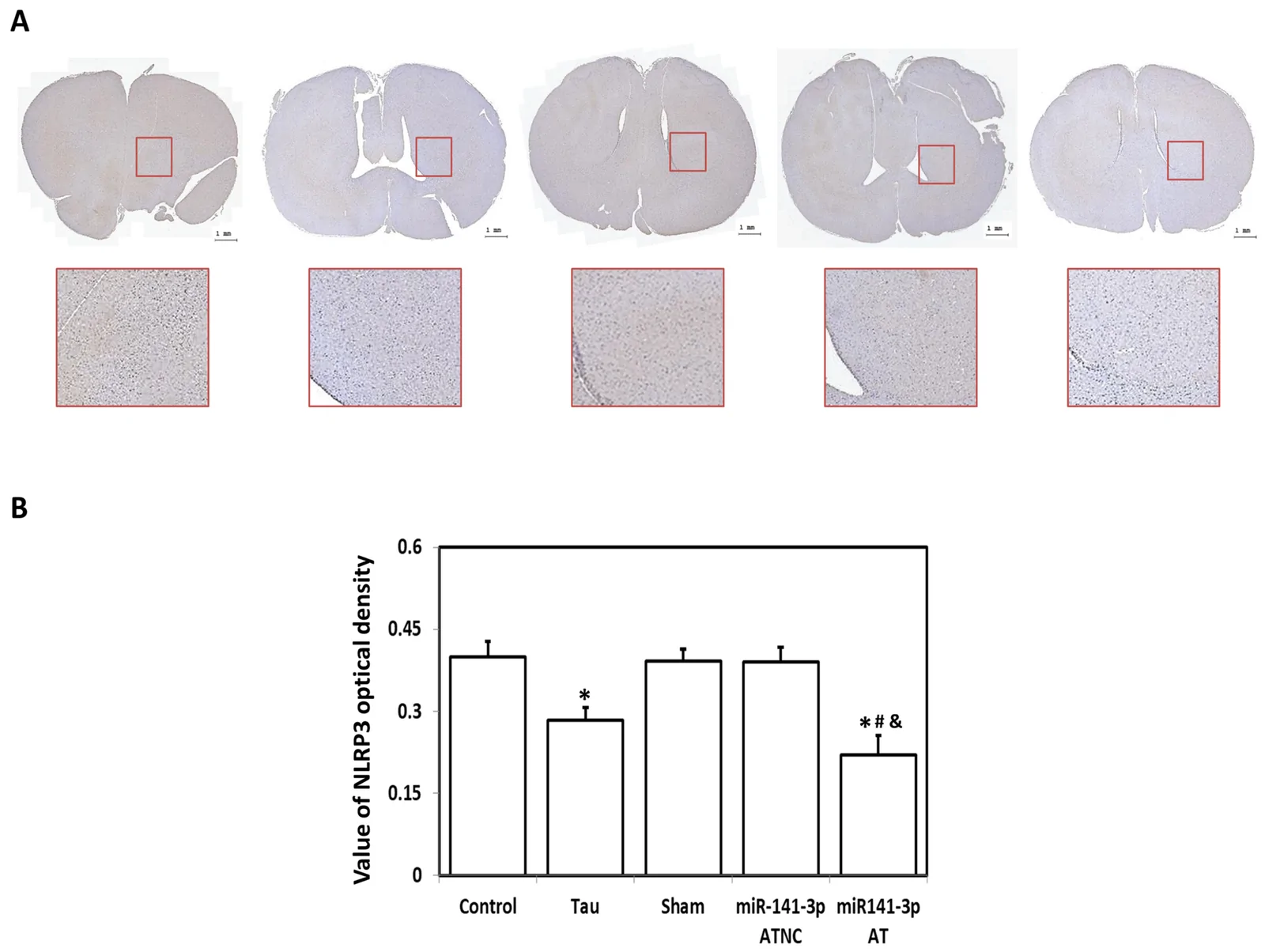
Immunohistochemical staining of NLRP3 in the striatum of SHRs. (**A**) Representative images of striatal sections immunohistochemically stained for NLRP3 in SHRs subjected to different treatments. Darker staining indicates stronger NLRP3 immunoreactivity. (**B**) Quantification of NLRP3-positive cells. Data are presented as the mean ± S.D. The symbols *, #, and & indicate significant differences compared with the Control, Sham, and miR-141-3p ATNC groups, respectively. Control: fed the Cho diet; Tau: fed 45 mM taurine; Sham: fed the Cho diet; miR ATNC: injected with the miR-141-3p antagomir negative control; miR AT: injected with the miR-141-3p antagomir.

To verify the roles of miR-141-3p on inflammation in the striatum of SHRs, various inflammatory signal pathways, including TNF-α/TNFR1 and TLR4/MyD88 routes, were investigated. Significantly reduced TNF-α, TNFR1, TLR4, and MyD88 proteins were detected in the striatum of SHRs fed with taurine compared to those from the Control group. Significantly lower levels of TNF-α, TNFR1, TLR4, and MyD88 proteins were also observed in the striatum of SHRs treated with a striatal stereotaxic injection of miR-141-3p AT compared to those from the Control, Sham, and miR-141-3p ATNC groups, respectively (Figure 2A,B). We also examined downstream molecules involved in inflammasome pathways. Significantly reduced levels of NF-κB, NLRP3, cleaved caspase-1, and cleaved IL-18 proteins were detected in the striatum of SHRs fed with taurine compared to those from the Control group. Consistently, significantly lower levels of NF-κB, NLRP3, cleaved caspase-1, and cleaved IL-18 were observed in the striatum of SHRs treated with miR-141-3p AT compared to those from the Control, Sham, and miR-141-3p ATNC groups, respectively (Figure 3A,B). Moreover, we performed immunohistochemistry (IHC) to confirm inflammasome involvement. NLRP3-positive signals, a marker of inflammasome activation, were significantly reduced in the striatum of SHRs fed taurine compared with those in the Control and Sham groups. Likewise, NLRP3-positive signals were significantly reduced in SHRs treated with striatal stereotaxic injection of miR-141-3p AT compared with those in the miR-141-3p ATNC group (Figure 4A,B).

### 2.3. Administration of miR-141-3p Antagomir Reduces IL-17A Level in the Striatum of SHRs

**Figure 5:**
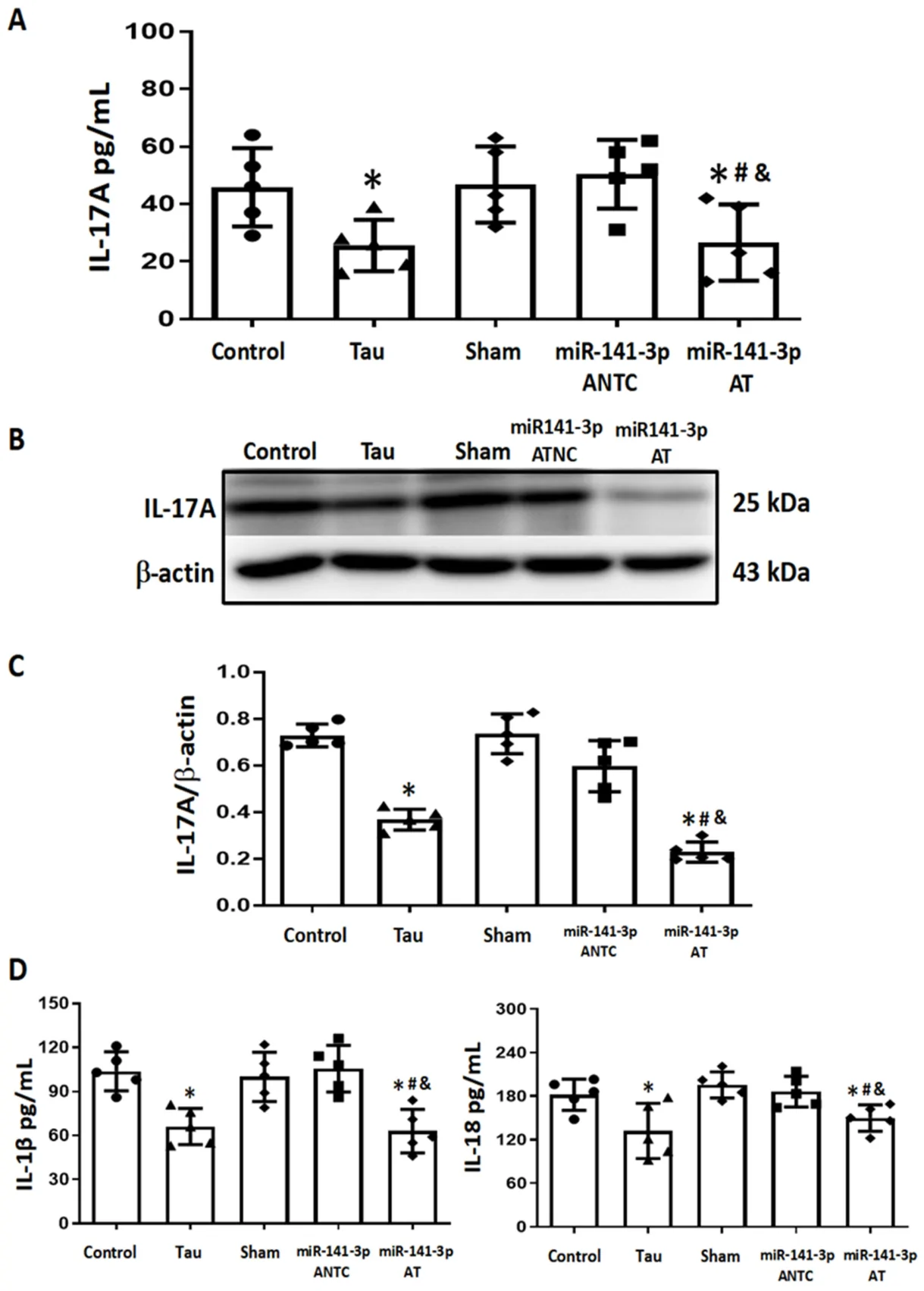
The levels of IL-17A, IL-1β, and IL-18 in the striatum of SHRs. (**A**) IL-17A concentration in the striatum of SHRs subjected to different treatments. (**B**) Expression of IL-17A protein in the striatum of SHRs subjected to different treatments. (**C**) Relative IL-17A protein levels normalized to β-actin. (**D**) IL-1β and IL-18 concentrations in the striatum of SHRs subjected to different treatments. Data are presented as the mean ± S.D. The symbols *, #, and & indicate significant differences compared with the Control, Sham, and miR-141-3p ATNC groups, respectively. Control: fed the Cho diet; Tau: fed 45 mM taurine; Sham: fed the Cho diet; miR ATNC: injected with the miR-141-3p antagomir negative control; miR AT: injected with the miR-141-3p antagomir.

To investigate the effects of miR-141-3p AT on IL-17A expression, an important pathological indicator in ADHD, in the striatum of SHRs, we performed ELISA and immunoblotting. A significantly lower concentration of IL-17A was detected in the striatum of SHRs fed with taurine compared to those from the Control group (Figure 5A). Similar findings were observed, with SHRs treated with striatal stereotaxic injection of miR-141-3p AT showing significantly lower IL-17A concentrations in the striatum than those in the Control, Sham, and miR-141-3p ATNC groups (Figure 5A). Consistent results were obtained by immunoblot analysis, which showed that SHRs fed taurine or treated with miR-141-3p AT exhibited significantly lower IL-17A levels in the striatum than those in the Control and Sham groups and the miR-141-3p ATNC group, respectively (Figure 5B,C). Similarly, the upstream mediators of IL-17A, IL-1β and IL-18, showed significantly lower striatal concentrations in SHRs treated with miR-141-3p AT than in the Control, Sham, and miR-141-3p ATNC groups (Figure 5D).

### 2.4. Administration of miR-141-3p Antagomir Increases Spontaneous Alternations in SHRs

**Figure 6:**
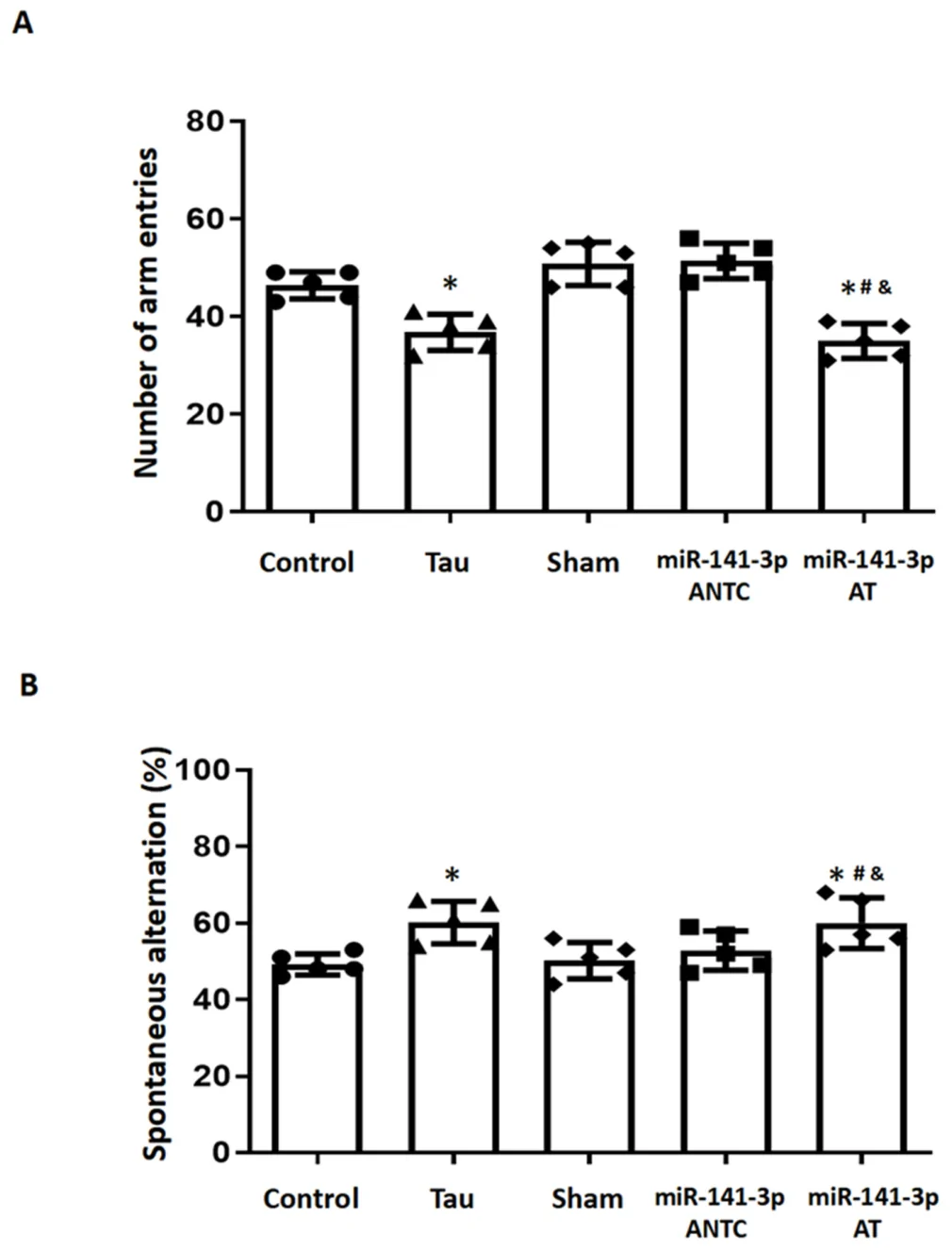
Arm entry and spontaneous alternation in SHRs. (**A**) Arm entry and (**B**) spontaneous alternation in SHRs from different treatment groups (*n* = 5 per group). Data are presented as the mean ± S.D. The symbols *, #, and & indicate significant differences compared with the Control, Sham, and miR-141-3p ATNC groups, respectively. Control: fed the Cho diet; Taurine: fed 45 mM taurine; Sham: fed the Cho diet; miR ATNC: injected with the miR-141-3p antagomir negative control; miR AT: injected with the miR-141-3p antagomir.

To verify the influence of miR-141-3p AT on working memory in SHRs, a three-arm Y-maze test was conducted to detect arm entry and spontaneous alternation. In SHRs treated with taurine, a significantly lower number of arm entries was observed compared to those from the Control group (Figure 6A). Similar results were observed in SHRs with striatal stereotaxic injection of miR-141-3p AT compared with those from the Control, Sham, and miR-141-3p ATNC groups (Figure 6A). Additionally, a significantly higher percentage of spontaneous alternation was observed in SHRs fed with taurine and those with striatal stereotaxic injection of miR-141-3p AT compared to those from the Control, Sham, and miR ATNC groups, respectively (Figure 6B).

## 3. Discussion

Stress during early life is associated with an increased prevalence of neurological disorders, including autism, attention-deficit/hyperactivity disorder (ADHD), schizophrenia, and depression [35]. Notably, chronic inflammation and oxidative stress can impair microglial function and may contribute to ADHD-related symptoms through persistent T cell-mediated neuroinflammation, neuronal oxidative damage, and disruption of normal brain function [36,37]. Inflammasomes are multiprotein complexes of the innate immune system that regulate caspase-1 activation and promote inflammatory responses. The NLRP3 inflammasome is one of the most extensively studied inflammasome components in microglia and promotes the release of various inflammatory cytokines upon activation, thereby contributing to neuroinflammatory processes in the central nervous system [38,39]. In the present study, miR-141-3p antagomir treatment was associated with significantly decreased levels of several inflammation- and inflammasome-related molecules involved in the TLR4/NF-κB/NLRP3, TNF-α/NF-κB/NLRP3, and IL-1β/IL-18/IL-17A signaling pathways in the striatum of SHRs. These findings suggest that inhibition of miR-141-3p may be associated with reduced neuroinflammatory and inflammasome-related responses and improved ADHD-like behavioral outcomes. However, the present study did not directly validate miR-141-3p downstream targets or establish a causal relationship between miR-141-3p inhibition, these inflammatory pathways, and behavioral changes. The observed alterations in TLR4/NF-κB/NLRP3, TNF-α/NF-κB/NLRP3, and IL-1β/IL-18/IL-17A signaling should be interpreted as associations rather than definitive evidence of pathway-specific mediation.

Although miR-141-3p has been implicated in diverse biological processes, including neural development, differentiation, apoptosis, and cancer [40,41,42,43,44], its role in ADHD remains largely unexplored. Emerging evidence suggests that miR-141-3p is involved in neuronal function and cognitive processes. Dysregulation of miR-141-3p has been associated with neuronal injury and functional deficits in neurological disease models [45], and miR-141-3p has also been implicated in neural stem cell proliferation, differentiation, and neurogenesis [34]. Notably, NRXN1, a synaptic adhesion molecule involved in synapse formation and cognitive function, has been implicated in ADHD pathophysiology [46]. Importantly, a recent study using spontaneously hypertensive rats (SHRs), an established animal model of ADHD, demonstrated that the MALAT1–miR-141-3p/200a-3p–NRXN1 axis was associated with learning and memory deficits [47], providing a direct rationale for investigating miR-141-3p in ADHD-related cognitive dysfunction. In parallel, miR-141-3p may regulate neuroinflammation. In a rat model of bacterial meningitis, miR-141-3p overexpression attenuated astrocyte activation and reduced TNF-α, IL-1β, and IL-6 production by targeting HMGB1 [48]. Although this evidence comes from a neurological inflammatory disease model rather than an ADHD model, it suggests that miR-141-3p can modulate neuroinflammatory responses. Given the growing evidence implicating neuroinflammation and inflammatory signaling pathways, including TLR4/NF-κB/NLRP3, in ADHD-related cognitive and behavioral abnormalities, these converging findings suggest that miR-141-3p may represent a molecular link between neuroinflammation and cognitive dysfunction in ADHD. Consistent with this possibility, taurine treatment in the present study was associated with altered miR-141-3p expression and improvements in cognitive and inflammatory outcomes, suggesting a potential role for miR-141-3p in taurine’s effects. However, whether miR-141-3p directly regulates the inflammatory pathways identified in this study and whether such regulation causally contributes to ADHD-related cognitive dysfunction remain to be determined. Future studies involving direct manipulation of miR-141-3p and appropriate rescue experiments will be necessary to establish the causal role of miR-141-3p in the neuroinflammatory and cognitive phenotypes associated with ADHD.

Interleukin-17A (IL-17A), a pro-inflammatory cytokine primarily produced by T helper 17 (Th17) cells, has been implicated in autoimmune, neuroinflammatory, and other pathological conditions [49]. Emerging evidence suggests that IL-17A may also influence neuronal function and cognitive processes. Genetic deletion of IL-17A improved behavioral outcomes and reduced neuroinflammation-related gene expression, including IL-1β, IL-6, NOX2, and NOX4, in sevoflurane-exposed neonatal mice [50]. IL-17A has also been shown to impair synaptic plasticity and contribute to cognitive deficits in an experimental model of multiple sclerosis [51]. Moreover, IL-17A blockade attenuated microglial activation, neuroinflammation, and cognitive decline in an aging mouse model [52]. Although the specific role of IL-17A in ADHD remains unclear, alterations in inflammatory and immune-related factors in individuals with ADHD suggest a potential contribution of immune dysregulation to ADHD pathophysiology [33,53,54]. The rationale for examining IL-17A in the present study is further supported by its association with the inflammatory pathways investigated herein. IL-17A can activate NF-κB-dependent inflammatory signaling and promote pro-inflammatory cytokine production. In particular, IL-17A has been reported to induce TNF-α production by microglia through the TLR4/NF-κB pathway and to promote NLRP3 inflammasome activation through NF-κB-related mechanisms, thereby increasing IL-1β production [53,55]. Thus, IL-17A may be functionally associated with the TLR4/NF-κB/NLRP3 and TNF-α/NF-κB/NLRP3 pathways examined in the present study, providing a potential link between IL-17A-associated neuroinflammation and cognitive dysfunction. Consistent with this rationale, we observed changes in IL-17A following miR-141-3p inhibition, suggesting an association between miR-141-3p and IL-17A-related inflammatory responses. However, whether miR-141-3p directly regulates IL-17A or these inflammatory pathways remains unclear and warrants further investigation.

Taurine is a sulfur-containing amino acid with diverse physiological functions, including osmoregulation, energy metabolism, immune modulation, antioxidant activity, and maintenance of neuronal function [56,57,58]. Beyond these well-established biological effects, taurine has also been shown to modulate gene expression involved in cell-cycle regulation, intracellular signaling, metabolism, cell survival, protein synthesis, and aging [59,60]. More recently, growing evidence suggests that taurine exerts part of its neuroprotective effects by regulating microRNAs (miRNAs). For example, taurine increases miR-21 and decreases miR-146 expression in SH-SY5Y neuroblastoma cells, thereby increasing matrix metalloproteinase-9 (MMP-9), an amyloid-β-degrading enzyme, and suggesting a potential mechanism underlying its neuroprotective effects [61,62]. Consistent with this concept, taurine administration has been shown to attenuate ADHD-like behaviors in spontaneously hypertensive rats (SHRs) by suppressing striatal miR-200b-3p expression [29]. In the present study, we further showed that taurine significantly reduced miR-141-3p expression, raising the possibility that modulation of multiple miRNAs may be associated with the beneficial effects of taurine in this ADHD model. Notably, taurine treatment and miR-141-3p antagomir treatment produced similar behavioral and anti-inflammatory effects. However, these findings do not establish that the behavioral or anti-inflammatory effects of taurine are mediated specifically through miR-141-3p. These findings indicate that miR-141-3p inhibition is associated with behavioral and anti-inflammatory effects similar to those observed with taurine treatment; however, whether these effects of taurine are mediated through miR-141-3p remains unclear. Nevertheless, the causal relationship between taurine, miR-141-3p, and the observed behavioral and inflammatory changes remains to be determined. A limitation of the present study is that we did not directly determine whether the behavioral and anti-inflammatory effects of taurine are mediated through miR-141-3p. It is also unknown whether miR-141-3p and miR-200b-3p regulate common or distinct molecular pathways involved in ADHD pathophysiology. Future studies should investigate their downstream targets and signaling networks and determine whether the effects of taurine are dependent on miR-141-3p and whether combined modulation of these miRNAs produces additive or distinct effects. Such studies may provide further insight into the molecular mechanisms underlying the neuroprotective effects of taurine and the contribution of miRNA regulation to ADHD pathophysiology.

The Y-maze spontaneous alternation test was selected to assess working memory and attentional function, which are relevant components of executive function that may be impaired in ADHD [63,64]. Impaired spontaneous alternation has also been reported in SHR-based models of ADHD, supporting its relevance to ADHD-related cognitive dysfunction [65,66]. Therefore, the changes in spontaneous alternation observed in the present study primarily reflect alterations in working memory-related cognitive function and may represent the cognitive component of the ADHD-like phenotype in SHRs. However, spontaneous alternation in the Y-maze does not directly assess the core behavioral symptoms of hyperactivity or impulsivity. Thus, the present behavioral findings should be interpreted primarily in the context of ADHD-related cognitive dysfunction, and additional behavioral paradigms specifically assessing locomotor hyperactivity and impulsivity will be necessary to comprehensively characterize the broader ADHD-like phenotype.

Several limitations of the present study warrant careful consideration when interpreting the findings. First, we performed stereotactic injection only in the left striatum. Although this approach enabled localized manipulation of miR-141-3p, unilateral intervention may not fully reflect bilateral striatal function. Second, we evaluated taurine intervention and miR-141-3p antagonist treatment independently, without a combined treatment group; therefore, we could not determine potential synergistic or additive effects between taurine and miR-141-3p inhibition. In addition, the study did not include a PBS plus transfection reagent control group; therefore, the potential inflammatory effects of the transfection reagent itself cannot be completely excluded. The possibility of off-target effects of the miRNA antagonist also cannot be ruled out, as the specificity of the antagomir and its delivery efficiency to the brain were not directly examined. Finally, the relatively small sample size (n = 5 per group) and the lack of an a priori power analysis may have limited the study’s statistical power. Future studies incorporating bilateral intervention, combined taurine and miR-141-3p inhibition, appropriate vehicle controls, validation of antagomir specificity and delivery efficiency, and larger sample sizes with appropriate power calculations are warranted to further validate and strengthen these findings.

Finally, several challenges associated with miRNA-based therapeutics should be carefully considered before translating miR-141-3p antagomir (AT) into a potential treatment for ADHD. Although substantial progress has been made in the preclinical development of miRNA-based therapies, only a limited number of candidates have advanced to clinical trials for diseases such as advanced solid tumors, Alport syndrome, type 2 diabetes with non-alcoholic fatty liver disease, heart failure, keloid disorder, chronic hepatitis C virus infection, and neurodegenerative disorders [67,68]. However, several of these clinical trials have been terminated or faced significant challenges because of toxicity, insufficient target specificity, immunogenicity, off-target effects, inefficient delivery, and a lack of reliable biomarkers to evaluate therapeutic efficacy [50,67,68]. Therefore, before miR-141-3p AT can be considered for clinical application in ADHD, its biological functions, downstream regulatory networks, long-term safety, delivery efficiency, and potential ethical considerations require comprehensive investigation.

## 4. Methods

### 4.1. Animals and Treatments

A total of 25 male spontaneously hypertensive rats (SHR/NCrlCrlj; SHRs, an animal model of ADHD) aged 3 weeks and weighing 38–42 g and 10 male Wistar Kyoto (WKY) rats aged 3 weeks and weighing 35–39 g were obtained from BioLASCO Taiwan Co., Ltd., Taipei City, Taiwan, to investigate the effects of miR-141-3p antagomir (AT). The animals were housed in a temperature- (22–24 °C), humidity- (50–60%), and light-controlled facility (12-h light/dark cycle) with free access to water and chow (Lab Diet 5001; PMI Nutrition International, St. Louis, MO, USA) for 1 week. The animals were then randomly divided into seven groups (*n* = 5 per group): WKY, WKY + Tau, Control (SHRs), Tau (SHRs + Tau), Sham, miR-ATNC (miR-141-3p antagomir negative control), and miR-AT (miR-141-3p antagomir) groups. Animals in the Tau group were provided with a taurine-supplemented diet as described previously [29], whereas rats in the other groups were fed a standard chow diet until sacrifice. At 5 weeks of age, rats in the Sham, miR-ATNC, and miR-AT groups received stereotactic injections into the striatum under anesthesia induced by intraperitoneal injection of thiopental sodium (40 mg/kg body weight). A three-arm Y-maze test was performed on the day before sacrifice. If a rat lost more than 15–20% of its body weight or exhibited an arched back, timidity, or reduced activity, the experiment was terminated humanely, and the animal was euthanized to minimize suffering. At 8 weeks of age, the rats were euthanized by carbon dioxide (CO_2_) asphyxiation. The CO_2_ flow rate was set at 50% of the chamber volume per minute. After visual confirmation that the rats had stopped breathing, CO_2_ flow was maintained for an additional minute to ensure death. Striatal tissues were collected from all animals and stored at −80 °C until further analysis. The Institutional Animal Care and Use Committee of Chung Shan Medical University approved and supervised all experimental protocols (IACUC approval number: 2136).

### 4.2. Quantitative Real-Time PCR (RT-qPCR)

The levels of miR-141-3p were measured using quantitative real-time PCR (RT-qPCR), as described previously [69]. Briefly, total RNA was extracted from the striatum of SHRs using an miRNeasy Kit (Cat. #217604; Qiagen, Germantown, MD, USA). The Applied Biosystems StepOnePlus Real-Time PCR System and miRCURY LNA SYBR^®^ Green PCR Kit (Cat. #339345; Qiagen, Germantown, MD, USA) were used to detect miR-141-3p levels in rat striatal tissues. The primers used in this study were designed as described previously [47]. The forward and reverse primers for miR-141-3p were 5′-ACACTCCAGCTGGGCATCTTCCAG-3′ and 5′-CTCAACTGGTGTCGTGGAGTCGGC-3′, respectively. The forward and reverse primers for β-actin were 5′-CCCATCTATGAGGGTTACGC-3′ and 5′-TTTAATGTCACGCACGATTTC-3′, respectively.

### 4.3. MicroRNA and Stereotaxic Injection

We performed striatal stereotaxic injection as described previously [70,71], with modifications. Eight-week-old spontaneously hypertensive rats (SHRs; 200–230 g) were anesthetized by intraperitoneal injection of sodium thiopental (40 mg/Kg body weight) and placed on a heating pad in a stereotaxic frame. We exposed the skull and positioned the head relative to bregma. A small burr hole was drilled above the left striatum, and 5 nmol of rat miR-141-3p antagomir (AT) or its negative control (ATNC) (BioLion Technology Co., Ltd., Taipei, Taiwan) was dissolved in 1 µL of PBS and thoroughly mixed with 1 µL of HiPerFect transfection reagent (Cat. #301705; Qiagen, Germantown, MD, USA), resulting in a total injection volume of 2 µL. The mixture was injected into the left striatum at the following coordinates relative to bregma: anteroposterior (AP), +1.0 mm; mediolateral (ML), −2.5 mm; and dorsoventral (DV), −5 mm. The injection was performed using a 10-µL Hamilton syringe (Sigma-Aldrich, St. Louis, MO, USA) connected to a microinfusion pump (Stoelting Co., Wood Dale, IL, USA) at a rate of 1 µL/min. After injection, the needle was left in place for 5 min before being slowly withdrawn to minimize backflow. We then sutured the incision and disinfected it with iodine. We established the stereotaxic coordinates based on our previous dye-injection validation and published stereotaxic studies in SHRs [70,71]. For these experiments, we therefore did not repeat injection-site verification. Each experimental group included five rats, and n = 5 biological replicates were used for all experiments. Investigators responsible for animal allocation were aware of group assignments, whereas those conducting behavioral tests and quantifying Western blot, ELISA, and IHC data were blinded to group identities. Group assignments were disclosed only after completion of the respective analyses.

### 4.4. Enzyme-Linked Immunosorbent Assay (ELISA)

The concentration of IL-17A, interleukin-1β (IL-1β), and interleukin-18 (IL-18) in the striatum of SHRs was measured using an ELISA kit obtained from MyBioSource (Cat. #MBS704126; MyBioSource, San Diego, CA, USA; Cat. #: BMS630, Invitrogen, Thermo Fisher Scientific, Waltham, MA, USA; Cat. #: KRC2341, Invitrogen, Thermo Fisher Scientific, Waltham, MA, USA). Briefly, rat striatal tissues were collected and homogenized, and tissue lysates were obtained by centrifugation. The IL-17A concentration was measured according to the manufacturer’s instructions.

### 4.5. Western Blot

Western blotting was performed to detect inflammation-related proteins as described previously [28,29]. Briefly, striatal tissues from SHRs were immersed in and homogenized with PRO-PREP™ buffer (iNtRON Biotechnology, Inc., Seongnam-si, Gyeonggi-do, Republic of Korea). Protein concentrations were determined using a modified Bradford assay. Protein samples were separated by sodium dodecyl sulfate–polyacrylamide gel electrophoresis (SDS-PAGE) and subsequently transferred onto nitrocellulose membranes (Amersham Biosciences, Piscataway, NJ, USA). The membranes were blocked with 5% nonfat milk overnight at 4 °C and subsequently incubated with primary antibodies against NLR family pyrin domain-containing 3 (NLRP3; cat. #A12694; ABclonal), NF-κB, TNF-α, TLR-4, caspase-1 (cat. #A0964; ABclonal Biotech Co., Ltd., Woburn, MA 01801, USA), MyD88, TNFR, IL-17A, and IL-18 (cat. #061115; MilliporeSigma, Burlington, MA, USA) or β-actin (cat. #MAB1501; Merck Millipore, Burlington, MA, USA) with gentle agitation for 6 h at 4 °C. After incubation with a horseradish peroxidase (HRP)-conjugated secondary antibody for 1 h, the antigen–antibody complexes were detected using Immobilon Western Chemiluminescent HRP Substrate (Millipore, Billerica, MA, USA) and an imaging analyzer (GE ImageQuant TL 8.1, GE Healthcare Life Sciences, Marlborough, MA USA).

### 4.6. Immunohistochemistry (IHC)

Immunohistochemistry (IHC) was performed to detect NLRP3 expression in the striatum of SHRs. After euthanizing the animals with carbon dioxide, striatal tissues were collected, fixed in 10% formalin, and embedded in paraffin wax. Subsequently, the embedded striatal tissues were sectioned into 5-µm-thick slices and incubated overnight with an antibody against NLRP3 (cat. #A12694; ABclonal). The sections were then quantified by measuring the mean integrated optical density using the TissueFAXS PLUS system (TISSUE GNOSTICS, Vienna, Austria).

### 4.7. The Y-Maze Test

A three-arm Y-shaped maze, illuminated at 200 lx, was used to test rats’ spatial learning and memory abilities, based on a previous study [29]. The three arms are arranged at 120° angles, and each measures 20 inches long, 4 inches wide, and 15 inches high. Each rat was considered to have entered an arm when all four paws were inside. Spontaneous alternation was defined as the rat entering all three arms in consecutive choices, forming overlapping triplet sets. The percentage of spontaneous alternation was calculated based on the following formula: (actual alternations/maximal alternations) × 100%. The maximum number of alternations was determined as the total number of arm entries minus two.

### 4.8. Statistical Analysis

Experimental data were analyzed using GraphPad Prism 5.0, and the results are presented as the mean ± standard deviation (SD). The expression of miR-141-3p between WKY rats and SHRs was compared using two-way ANOVA. A one-way ANOVA followed by Tukey’s post hoc test was used to evaluate the significance of the experimental treatments in SHRs. A *p*-value of less than 0.05 (*p* < 0.05) was considered statistically significant.

## 5. Conclusions

**Figure 7:**
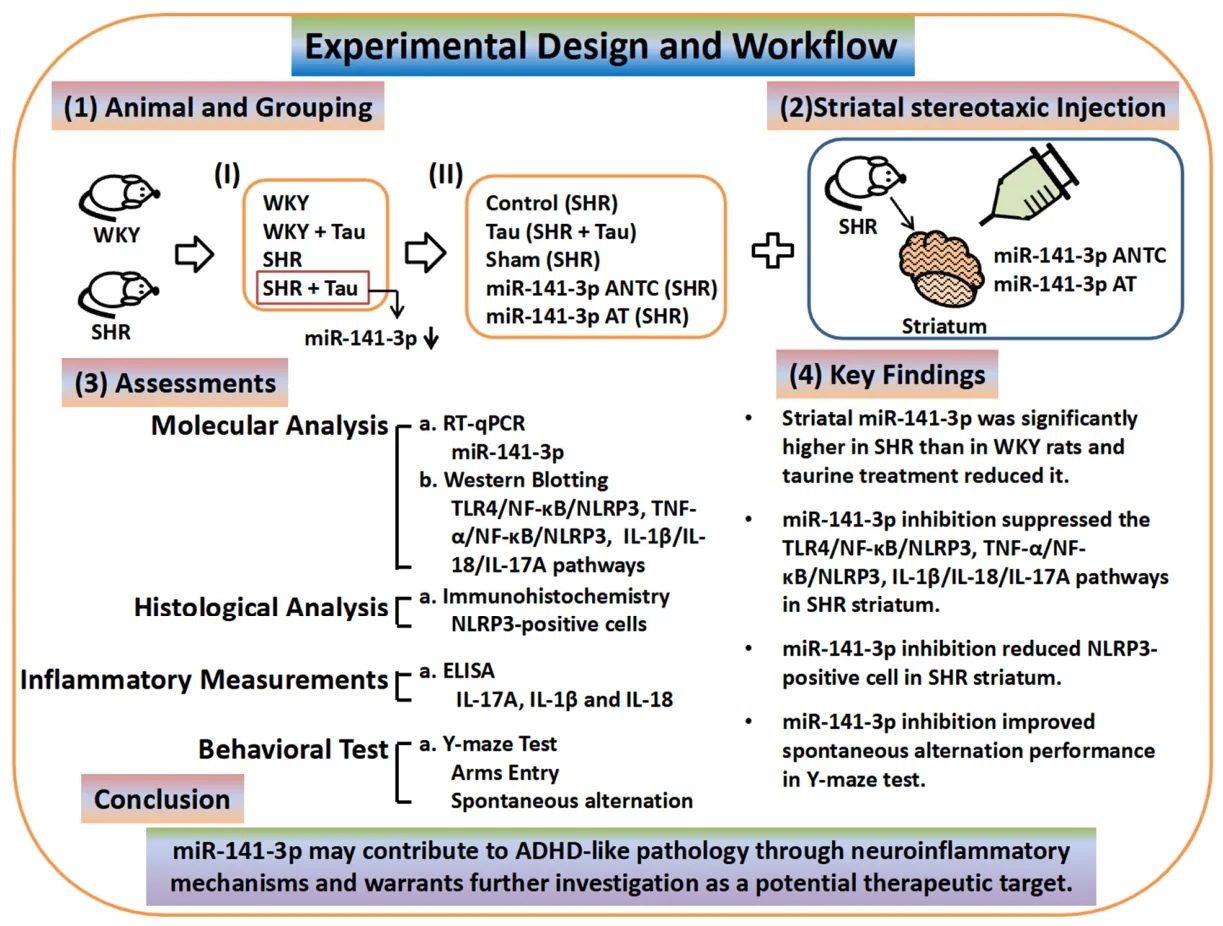
Schematic Overview of the Experimental Design and Workflow.

The present study provides preclinical evidence that inhibition of striatal miR-141-3p is associated with altered Y-maze spontaneous alternation, a measure related to working memory and exploratory behavior, in SHRs. This effect was accompanied by reduced levels of several neuroinflammatory markers and inflammatory mediators, including components of the TLR4/NF-κB/NLRP3, TNF-α/NF-κB/NLRP3, and IL-1β/IL-18/IL-17A pathways. Furthermore, taurine treatment reduced miR-141-3p expression, suggesting that miR-141-3p may contribute to taurine’s effects on cognition-related phenotypes in this ADHD model. Notably, the reduced number of arm entries following taurine or miR-141-3p antagomir administration may reflect changes in locomotor or exploratory activity and could influence the interpretation of spontaneous alternation (Figure 7). Therefore, these findings should not be interpreted as direct evidence of improvements in the core ADHD domains of inattention, hyperactivity, or impulsivity. Collectively, the findings suggest a potential association between miR-141-3p, neuroinflammatory signaling, and working memory-related behavior in SHRs. However, the present study does not establish a direct causal relationship or confirm that the observed behavioral effects are mediated specifically through these inflammatory pathways. Further studies involving direct target validation, rescue or overexpression approaches, behavioral paradigms assessing distinct ADHD-related domains, and additional animal models are warranted to clarify the mechanistic role and translational relevance of miR-141-3p.

## Data Availability

The original contributions presented in this study are included in the article. Further inquiries can be directed to the corresponding authors.
